# PrCRS: a prediction model of severe CRS in CAR-T therapy based on transfer learning

**DOI:** 10.1186/s12859-024-05804-8

**Published:** 2024-05-20

**Authors:** Zhenyu Wei, Chengkui Zhao, Min Zhang, Jiayu Xu, Nan Xu, Shiwei Wu, Xiaohui Xin, Lei Yu, Weixing Feng

**Affiliations:** 1 Intelligent Systems Science and Engineering College, Harbin Engineering University, Liaoyuan Street, Harbin, 150006 Heilongjiang Province People’s Republic of China; 2 School of Chemical and Molecular Engineering, East China Normal University, Zhongshan North Street, Shanghai, 200000 People’s Republic of China; 3 Shanghai Unicar-Therapy BioMedicine Technology Co., Ltd, Shanghai, China

**Keywords:** CAR-T immunotherapy, CRS, Deep learning, Transfer learning, Platform

## Abstract

**Background:**

CAR-T cell therapy represents a novel approach for the treatment of hematologic malignancies and solid tumors. However, its implementation is accompanied by the emergence of potentially life-threatening adverse events known as cytokine release syndrome (CRS). Given the escalating number of patients undergoing CAR-T therapy, there is an urgent need to develop predictive models for severe CRS occurrence to prevent it in advance. Currently, all existing models are based on decision trees whose accuracy is far from meeting our expectations, and there is a lack of deep learning models to predict the occurrence of severe CRS more accurately.

**Results:**

We propose PrCRS, a deep learning prediction model based on U-net and Transformer. Given the limited data available for CAR-T patients, we employ transfer learning using data from COVID-19 patients. The comprehensive evaluation demonstrates the superiority of the PrCRS model over other state-of-the-art methods for predicting CRS occurrence. We propose six models to forecast the probability of severe CRS for patients with one, two, and three days in advance. Additionally, we present a strategy to convert the model's output into actual probabilities of severe CRS and provide corresponding predictions.

**Conclusions:**

Based on our findings, PrCRS effectively predicts both the likelihood and timing of severe CRS in patients, thereby facilitating expedited and precise patient assessment, thus making a significant contribution to medical research. There is little research on applying deep learning algorithms to predict CRS, and our study fills this gap. This makes our research more novel and significant. Our code is publicly available at https://github.com/wzy38828201/PrCRS. The website of our prediction platform is: http://prediction.unicar-therapy.com/index-en.html.

## Background

Chimeric antigen receptor T (CAR-T) cell therapy represents a novel approach to immune-targeted treatment for malignant tumors, particularly revolutionizing the management of hematological malignancies. Notably, CAR-T cell therapy has demonstrated unprecedented efficacy in relapsed/refractory (R/R) B-cell acute lymphoblastic leukemia (B-ALL), non-Hodgkin's lymphoma (NHL), and multiple myeloma (MM) [1]. However, CAR-cell therapy is associated with a potentially life-threatening complication known as cytokine release syndrome (CRS). CRS, characterized by systemic inflammatory response triggered by the hyperactivation of CAR-T cells and endogenous immune cells (e.g., macrophages and dendritic cells), represents the most prevalent adverse event. Therefore, accurate prediction of the onset of severe CRS in CAR-T cell therapy holds paramount importance.

Deep learning is currently a highly popular technique with extensive applications and significant value in the biomedical industry. Its remarkable success in computer vision, speech recognition, and natural language processing (NLP) has led to its widespread adoption in DTI and other predictive tasks [2]. Use deep learning to interpret information about proteome sequences [3], and use deep learning models to predict antigenic peptides [4]. However, there is a lack of deep learning applications specifically focused on cytokine storm prediction. Given the inherent ability of deep learning models to learn automatically, certain methods such as transfer learning have been employed for DTI analysis. Transfer learning is a technique that leverages pre-trained models on one dataset to make predictions on different but related datasets, thereby enabling the development of more generalized models [5]. Consequently, this approach has garnered significant attention in the field of bioinformatics, encompassing research focused on unraveling biological system degradation [6]. Single-cell RNA sequencing [7], drug sensitivity prediction [8], and patient response estimation [9] are key applications in drug discovery. Transfer learning is predominantly employed in three domains, namely molecular characteristics and activity prediction (including DTI), molecular generation, and structure-based virtual screening [10]. Transfer learning serves as a fundamental approach to address the inherent challenge of limited training data in machine learning development [10]. For instance, in molecular generation models, it is common practice to pre-train models on extensive datasets such as Chemical European Molecular Biology (ChEMBL) [11], followed by fine-tuning the model using smaller target datasets to generate specific functional molecules. Subsequently, the knowledge gained from the initial model is leveraged [12]. The obtained parameters serve as initializations for the second model, and transfer learning can address the issue of data loss by fine-tuning a pre-trained model trained on extensive datasets [12].

The Transformer model, a widely used architecture developed by Vaswani [13], is solely based on the attention mechanism, eliminating the need for loops and convolutions. Schwaller and Lee's team successfully applied the Molecular Transformer model to accurately predict chemical reactions while considering uncertainty calibration [14]. In the realm of pharmaceutical chemistry, Lee employed the Transformer model to integrate reaction prediction and inverse synthesis, aiming for a comprehensive approach [15]. To enhance analysis accuracy, prediction precision, and establish a more generalized model, this study introduces transfer learning into the Transformer framework [16–19]. Specifically, we construct a model named PrCRS based on the Squeezeformer architecture [20].

In this study, we propose PrCRS, a novel multi-label prediction model for identifying severe CRS based on Transformer and multi-head self-attention mechanism. Firstly, we pre-trained the COVID-19 dataset to equip the model with knowledge of relevant features through sufficient training. Furthermore, the acquired knowledge was effectively applied to a smaller dataset pertaining to our CAR-T therapy, resulting in improved accuracy of output predictions following training on limited data. To compare the performance with non-migration learning, we utilized the prediction results without migration as reference data.

## Methods and data

### Dataset

The migration data was derived from a cohort of 1801 patients. Suspected COVID-19 inpatients were identified using PCR, routine laboratory measurements, and ELLA cytokines, while concurrently documenting the severity of their condition at that particular time. The patients were identified by querying the individuals in the electronic database of the Department of Pathology who conducted both SARS-CoV-2 PCR-based detection and ELLA cytokine grouping. The cytokine data were obtained from the electronic database of the pathology department, while the clinical and demographic data were supplemented with information from the Mount Sinai data warehouse [21].

The training and testing data were obtained from a cohort of 202 patients diagnosed with B-ALL, comprising 62 pediatric individuals aged between 0 and 25 years, as well as 140 adult subjects aged between 25 and 75 years, who received treatment at the Affiliated Hospital of Suzhou Medical University in China. The comprehensive dataset encompassed various parameters including blood routine indices, biochemical markers, coagulation factors, and cytokine levels. Among the 202 patients diagnosed with B-ALL, a total of 154 patients (76.2%) experienced cytokine release syndrome (CRS), with the majority presenting with mild to moderate CRS (grade 1–2; 109/202; 54%), while a significant proportion developed severe CRS (grade 3–4; 45/202; 22.3%). When collating data, we strive to maintain data integrity and fill in missing data with the appropriate CRS rating. Specifically, we populate the operation using the median value of all of this data contained in the CRS level to which the data corresponds. For patients presenting with fever, the onset of cytokine release syndrome (CRS) is defined as the initial occurrence of a temperature ≥ 38.0 °C following CAR-T cell infusion, while CRS resolution is defined as the absence of fever or vasoactive drug administration for at least 24 h. Among these individuals, 131 experienced fever symptoms, whereas 23 patients developed CRS in the absence of fever symptoms. Detailed data can be found in Table 1.

**Table 1:** Baseline characteristics of the patients

Characteristics	Children (N = 62)	Adult (N = 140)	Total (N = 202)
*Sex*			
Female	22	76	98
Male	40	64	104
*Multiline treatment*			
Median	3	3	3
Range	1–9	0–13	0–13
*Number of recurrence*			
Median	1	0	0
Range	0–3	0–3	0–3
Transplant or not	13	30	43
*Extramedullary infiltration*			
Yes	2	11	13
No	50	112	162
*Protoplast*			
Median	3.25%	7.00%	5%
Range	0–86%	0–94.5%	0–94.5%
*Dead or not*			
Yes	9	22	31
No	53	94	147

### Architectural design

**Fig. 1:**
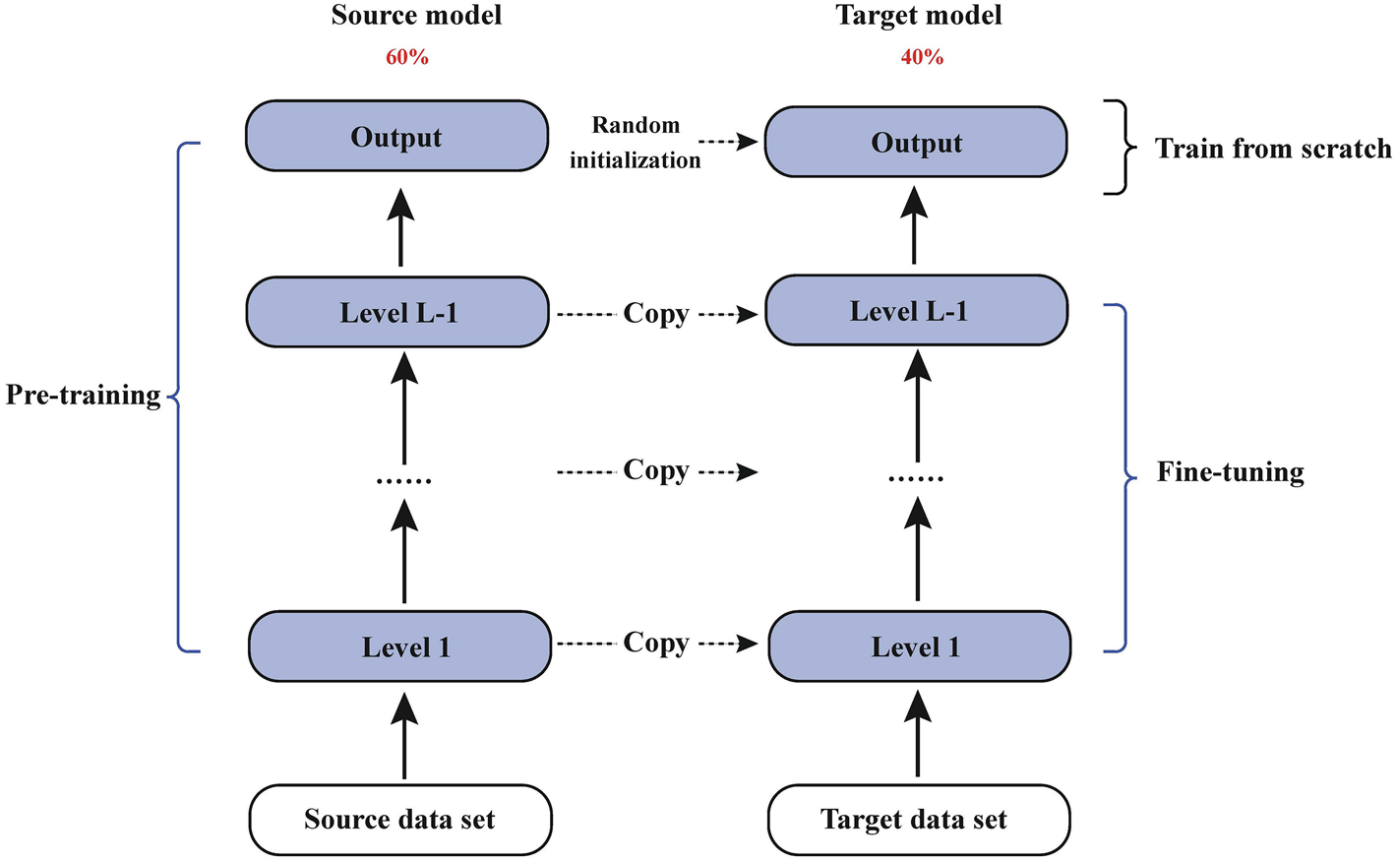
A transfer learning framework is employed, where the pre-training phase utilizes a COVID-19 dataset to achieve optimal results and model performance, with the COVID-19 dataset contributing 60% of the training data. Subsequently, a CAR-T dataset (accounting for 40%) is used for fine-tuning, leveraging the previously trained model. During this process, certain layers such as convolutional and Squeezeformer layers from the pre-training model are frozen

Due to limited availability of patient data on CAR-T cell therapy, this study employs transfer learning. For pre-training, we utilized a dataset related to novel coronavirus (COVID-19). Post-treatment, novel coronavirus also induces CRS reaction similar to that observed in CAR-T cell therapy; hence, this dataset was chosen as the migration data. Following extensive training, the source model acquires knowledge of relevant features from the data. The acquired knowledge is subsequently transferred to a smaller dataset pertaining to our CAR-T therapy through a Fine-tuning approach. Initially, the partial convolution layer and Squeezeformer of the pre-training model are kept frozen during training [20]. While certain layers of the model remain unchanged, the remaining layers and fully connected layers undergo training. The pre-training of the model is based on a data set that comprises 60% novel coronavirus data and 40% CAR-T data. This approach effectively harnesses the powerful generalization ability of deep neural networks while avoiding complex model design and lengthy training. The framework diagram for the model is presented in Fig. 1.

The robustness of the model architecture plays a crucial role in determining its overall performance. Among various options, convolutional neural network (CNN) is a popular choice for backbone model architecture. Initially, an end-to-end depth CNN model was explored; however, Transformer architecture has emerged as a promising alternative due to its attention mechanism that addresses long-term dependence between input and output while enabling parallel computing and reducing computational resource consumption. In this study, we adopt the PrCRS Transformer model as our basis. The proposed model, an enhanced version of Squeezeformer, incorporates a multi-head attention module to enable parallel computation in the encoder [13]. In the input layer, clinical factor data from COVID-19 and CAR-T treated patients are read and subsequently transformed into a fixed-size matrix through the embedding layer. The PrCRS layer employs a combination of U-Net and Transformer architectures to capture factor characteristics, with the resulting feature matrix fed into the classification layer for prediction.

**Fig. 2:**
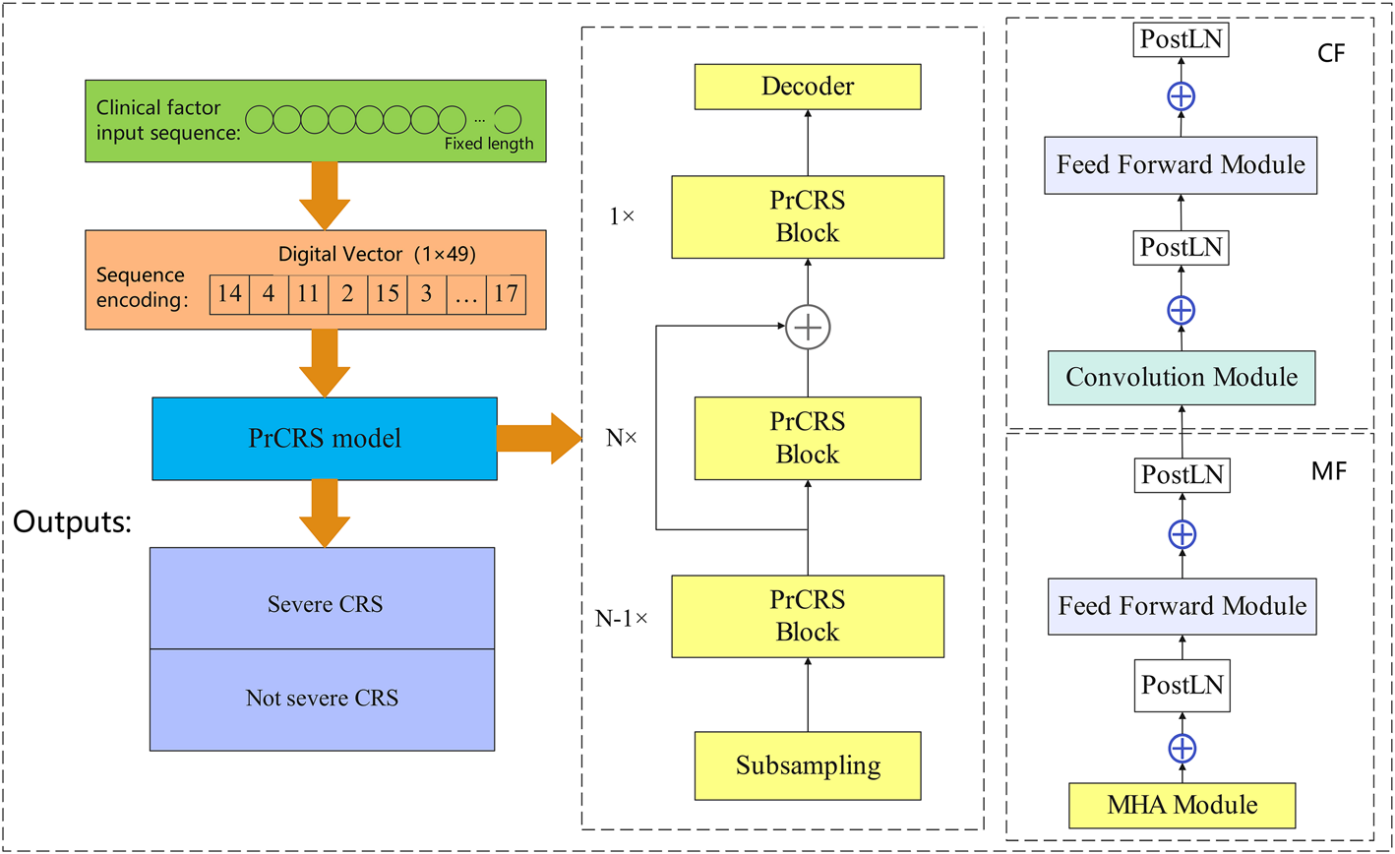
The PrCRS framework is structured as follows: Firstly, patient clinical factors are numerically encoded and then transformed into a fixed-size matrix using the embedding layer. Secondly, the PrCRS layer combines U-Net architecture with Transformer architecture to effectively capture factor characteristics. Thirdly, the model employs a multi-head self-attention mechanism to prioritize important discriminant features for predicting severe CRS occurrence. Finally, the resulting feature matrix is fed into the classification layer to score different levels and obtain accurate prediction results

PrCRS incorporates the U-Net architecture, enabling temporal compression of frame numbers in the intermediate layer and subsequent recovery in the final layer. Due to its utilization of the U-Net structure, our model demonstrates enhanced efficiency compared to other models with equivalent parameters. We employ a combination of Multi-head attention (MHA) + Feed forward network (FFN) + Convolutional module + FFN (MFCF). The architectural design of our model is illustrated in Fig. 2. Specifically, we propose a block structure that bears resemblance to the conventional Transformer [13, 22]. We further introduce a simplified block configuration where Multi-head attention (MHA) and convolution modules are sequentially followed by a feedforward module.

### U-Net architecture

The mixed attention-convolution structure of Conformer facilitates the capture of both global and local interactions. However, it is important to note that this operation incurs quadratic FLOPs complexity in relation to the input sequence length. In order to mitigate this additional overhead, we propose a method for calculating attention on a reduced sequence length using U-Net [23]. Inspired by the successful dense prediction architecture in computer vision, this study integrates the time U-Net structure. The compact network structure of U-Net reduces the number of network parameters and accelerates training speed, thereby mitigating overfitting risks and enhancing model generalization capabilities. Moreover, U-net introduces the Skip Connections structure, which establishes direct connections between the encoder and decoder feature maps in a non-linear manner, thereby preserving more spatial and contextual information. This enhancement significantly enhances segmentation accuracy and detail retention capabilities, as illustrated in Fig. 2. The up-sampling module employed in this study utilizes a higher sampling rate for processing embedded vectors. To the best of our knowledge, the work most closely related to our proposed time U-Net is [24]. In that paper, the U-Net architecture is integrated into a complete convolutional model for down-sampling sleep signals.

### Transformer module

The Conformer model was employed as a reference in our study [25]. The Conformer block encompasses a sequence of feedforward ('f'), multi-head attention ('m'), convolution ('c') layers, and another feedforward module ('f'). We denote this structure as FMCF. Notably, the convolution kernel exhibits a substantial size, endowing it with attention-like behavior by incorporating mixed global information. This stands in stark contrast to the convolution kernel commonly used in computer vision, which typically employs a small kernel size. Therefore, to enhance efficiency, we propose adopting the MF/CF structure, motivated by treating the convolution module as a local multi-head attention module. Furthermore, we opted to exclude the Macaron structure [26]. Due to its limited usage in the literature [13, 22, 27, 28], where multi-head attention modules and feedforward modules are more commonly employed. In summary, we simplified our architecture to resemble the standard Transformer network (Fig. 2), incorporating MHA and convolution modules followed by a feedforward module.

### Simplified layer normalization

LayerNorm is incorporated in the Conformer model, with both post-LayerNorm (postLN) applied between residual blocks and pre-LayerNorm (preLN) implemented within the residual connection. Although it is assumed that preLN remains stable during training and postLN contributes to improved performance [29], employing these two modules simultaneously results in redundant consecutive operations. In addition to architectural redundancy, the computational cost of LayerNorm can be significant due to its global reduction operation [30]. However, removing either preLN or postLN would result in unstable training and failed convergence. Therefore, it is crucial to incorporate a scaling layer when replacing the preLN component to enable network control over this weight. This concept is analogous to various training stability techniques employed in other domains. For instance, NF-Net [31] introduced adaptive scaling before and after the residual block to enhance training stability without normalization. Moreover, DeepNet [29] recently proposed incorporating untrained rule-based scaling into the skip connection to stabilize preLN in Transformers. Motivated by these findings, we have implemented a postLN-then-scaling approach to replace the preLN in all modules, as illustrated in Fig. 2. Consequently, our entire model now exclusively employs postLN. By substituting the redundant front layer normalization with scaled back layer normalization, we achieve zero reasoning cost and significantly reduce floating point operations (FLOP).

## Result

### Performance comparison

In order to establish an efficient model for predicting the cytokine release syndrome (CRS) of CAR-T therapy, we conducted a comparative analysis of various classical methods including CNN, Transformer, Squeezeformer, and our novel PrCRS model. In order to mitigate the impact of overfitting and enhance the model's generalization capability, we employed a fivefold cross-validation approach for optimal model selection. The classification performance of these models on the training set is presented in Table 2. Precision and recall, being crucial metrics in label classification evaluation, are employed to select a more optimal model. Our PrCRS model demonstrates superior performance compared to other models. Furthermore, deep learning-based models (Transformer and Squeezeformer) generally outperform classical models (CNN). Compared to models based on CNN, Transformer, and Squeezeformer, our PrCRS model achieves a minimum of 3% higher f1 score on the test set. Leveraging U-net and Transformer modules, our model optimizes the extracted feature matrix. Consequently, we employ the PrCRS model for predicting CRS in CAR-T therapy.

**Table 2:** Experimental findings

Model	CAR-T		
	Macro precision	Macro recall	Macro f1
CNN	0.5382	0.5884	0.5442
Transformer	0.6747	0.6806	0.6776
Squeezeformer	0.8613	0.7108	0.7640
PrCRS	0.9255	0.7482	0.8112

Comparative analysis of indices between the original and enhanced models in CAR-T data

Initially, we utilized the dataset comprising 1497 days of data from 202 patients diagnosed with acute B-lymphoblastic leukemia and treated with CAR-T therapy. For training, fine-tuning, and testing purposes, we considered a comprehensive set of 42 factors for all patients. The distribution of the dataset is allocated in a ratio of 6:2:2 for training, validation, and testing sets respectively. We adopted an experimental design based on the fivefold cross-validation method. The tags in our dataset were categorized into two levels: ‘severe CRS (≥ 3)’ and ‘non-severe CRS (< 3)’, thereby presenting a binary classification problem. During the training process, the average f1 score of the CNN model was observed to be 0.5177, while that of the Transformer model achieved a higher value of 0.6703. Subsequently, we conducted experiments using the Squeezeformer model. The CrossEntropyLoss() function was employed as the loss function, yielding optimal results. The Adam optimizer is employed to compute the output and update the parameters based on the gradient. Its average f1 score amounts to 0.7508. In view of the relatively large number of models, only the hyperparameter adjustment of our PrCRS model is described in detail here. The steps of the hyperparameter tuning method are as follows: First, with other hyperparameters fixed, only one hyperparameter is optimized in a specific interval, and the value of the hyperparameter with the best model effect is selected after the training is completed. Then, the selected value is taken as a fixed value, and the other hyperparameters are further optimized by the same method until the last hyperparameter is completely adjusted.

In our model, we initially utilized a COVID-19 dataset consisting of 1801 patients as the primary dataset, while the second dataset involved CAR-T therapy for patient treatment. The size of the first dataset exceeded that of the second one. We employed CrossEntropyLoss() as our loss function and Adam() as an optimizer. We adopted a method of individually adjusting one parameter while keeping the others fixed. We conducted experiments by testing multiple values within a specific range for each parameter and evaluated their impact on the model's performance. The value corresponding to the best model performance was selected as the optimal setting for that particular hyperparameter. This process was repeated for other hyperparameters as well. When training the target model, we utilized 150 epochs and set the batch size to 12. The learning rate is set to 0.001, and the final model achieves its peak performance at the 12th epoch. For fine-tuning, specific layers of both the pre-training model's convolution layer and Squeezeformer model were frozen. The final softmax layer was employed for classifying results as either “0” or “1”. Subsequently, the dataset underwent classification testing. The primary advantage of the PrCRS model lies in its ability to facilitate highly effective migration learning through the reuse of feature graph signatures acquired from the training model.

**Fig. 3:**
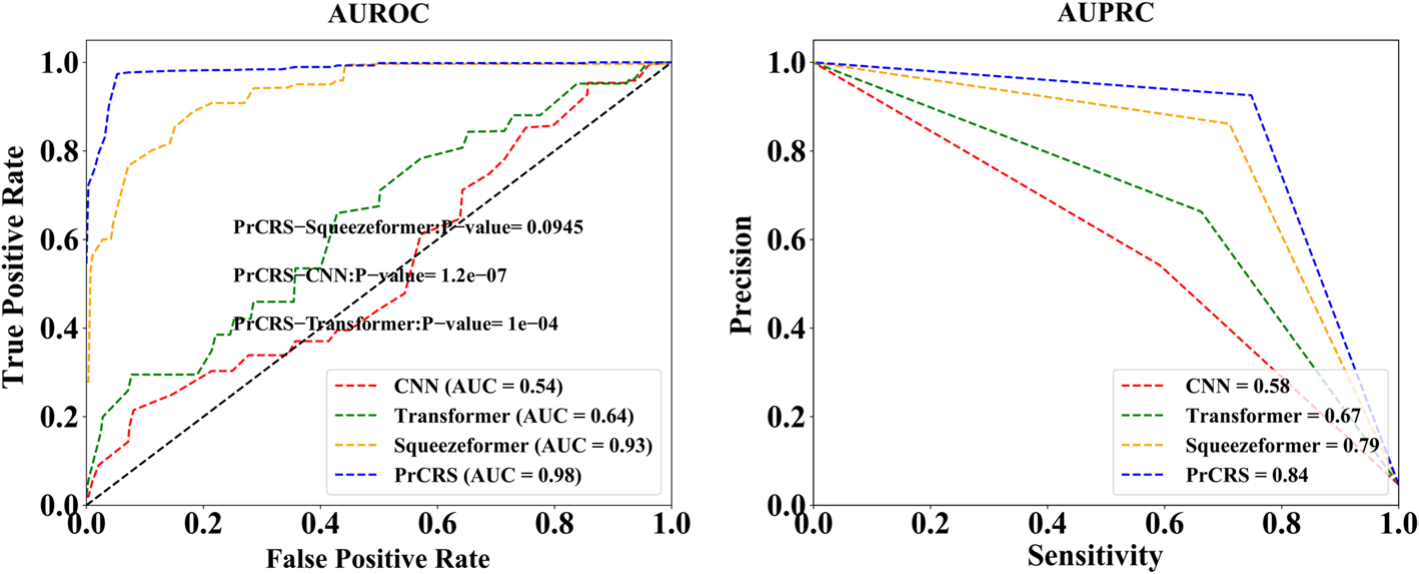
Experimental AUROC and AUPRC curves. AUROC curve representing the experiment. AUPRC curve representing the experiment

We conducted separate analyses for AUROC and AUPRC in each case, and the corresponding results are presented in Fig. 3. The AUROC and AUPRC outcomes are depicted as A and B in Fig. 3, respectively. It is evident that PrCRS exhibits comparable AUROC performance to squeezeformer, while surpassing Transformer and the conventional CNN model by over 40%. Furthermore, our model achieves the highest value in terms of AUPRC.

We separately show the effect of clinical factors on the model after removing a large item. The experiment effectively reveals the extent to which each detection item affects the model. The effect of the model is shown in Table 3.

**Table 3:** Experimental findings

Model	CAR-T		
	Macro precision	Macro recall	Macro f1
Del cytokines	0.6118	0.8877	0.6628
Del biochemical	0.6364	0.9727	0.7003
Del blood routine	0.9933	0.6667	0.7466
Del clotting and the rest	0.7949	0.7466	0.7685

The effect of each major clinical factor on the model effect was removed separately

**Fig. 4:**
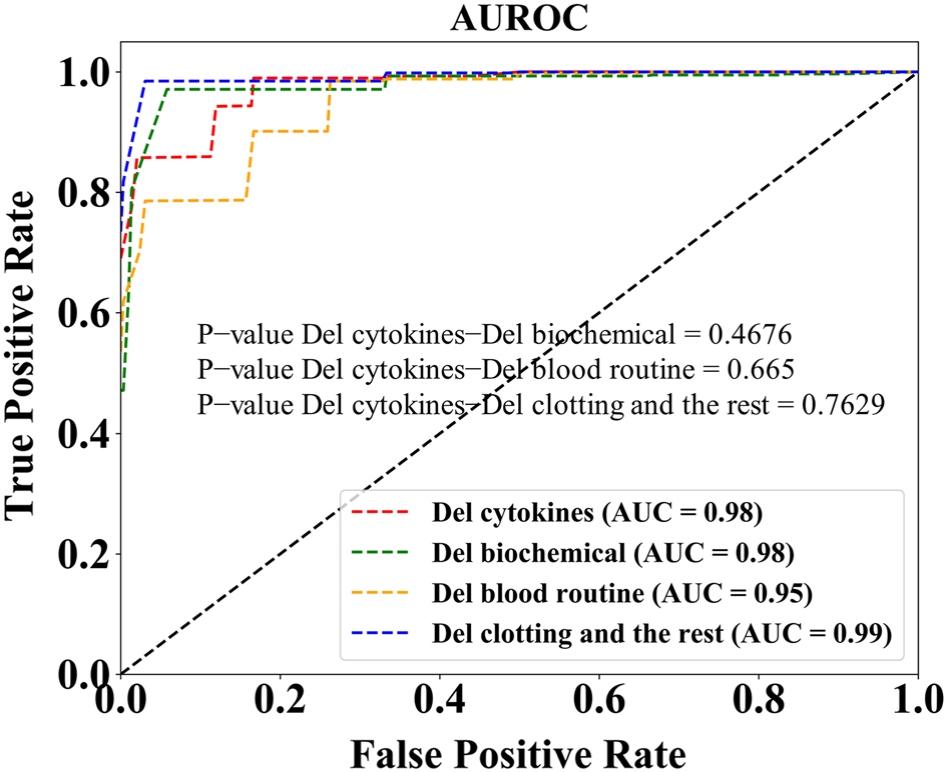
Experimental AUROC curves. The AUROC curve is depicted, along with the *p*-value obtained through a t-test for each term within it

The AUROC curve is depicted, along with the p-value obtained through a t-test for each term within it. We analyzed the AUROC in each case and show the corresponding results in Fig. 4. Figure 4 shows the results of AUROC and P values. Clearly, while there are differences between the items, they are not sufficiently different to produce competitive P-values. Although the difference is not significant from the P-value, there is still a significant difference from the F1 value. After the removal of cytokines, the F1 value was the lowest, indicating that cytokines played the most significant role in predicting the occurrence of severe CRS. Similarly, the effect was small when coagulation indexes and related factors were excluded. This is consistent with the actual results observed by doctors.

## Experimental validation

We retrained the model using data from the remaining 193 patients, and selected 9 individuals from the CAR-T dataset for individual verification. Daily images were drawn for analysis, as shown in Fig. 4. Our model assesses the severity of CRS in patients on a daily basis to validate its effectiveness. As the predictive power of the model diminishes with an increase in the number of days in advance, the results obtained for that specific day are considered optimal and most comparable. The actual prediction model forecasts patient conditions one, two, and three days ahead.

Out of the 9 patients, 5 exhibited severe cytokine release syndrome (CRS) with a grade equal to or higher than 3, while the remaining 4 did not experience such severity. In our CAR-T dataset, we define positive labels as cases with severe CRS (grade ≥ 3), negative labels as those without severe CRS (grade 0–2), and visually represent actual severe CRS using thick black vertical lines. According to the findings, it is evident that despite some individuals not exhibiting severe CRS, the model predicts a relatively high probability of occurrence, which aligns with real-world scenarios. This consistency arises from the fact that if a patient is falsely diagnosed with severe CRS when they do not actually have it, treatment response can be effectively controlled. However, in the event of its actual occurrence being falsely judged as not happening, there may be a potential threat to the patient's life. Therefore, to a certain extent, false positives are permissible and considered normal. Based on our model's outcomes, it has demonstrated excellent performance and can effectively assess severe CRS in patients.

Since the prediction probability of the model is influenced by its parameters, the output probability varies accordingly. Therefore, it does not accurately represent the actual probability of CRS but serves as a mere reference. To align with the actual clinical scenario and facilitate accurate medical decision-making, we employ a strategy to transform the model's prediction outcomes into probabilities representing the likelihood of patients developing severe CRS. In the first step, after training and testing, we use our own data to tune the parameters of the model and select the best model. Next, the test set is predicted using the best model, and the probability of severe CRS occurrence for each sample corresponds one-to-one to the original CRS grade label. When done, they are arranged in descending order of probability, and the CRS rank label order is adjusted accordingly. The sorted probability and CRS label sequence are then used as a baseline.

**Fig. 5:**
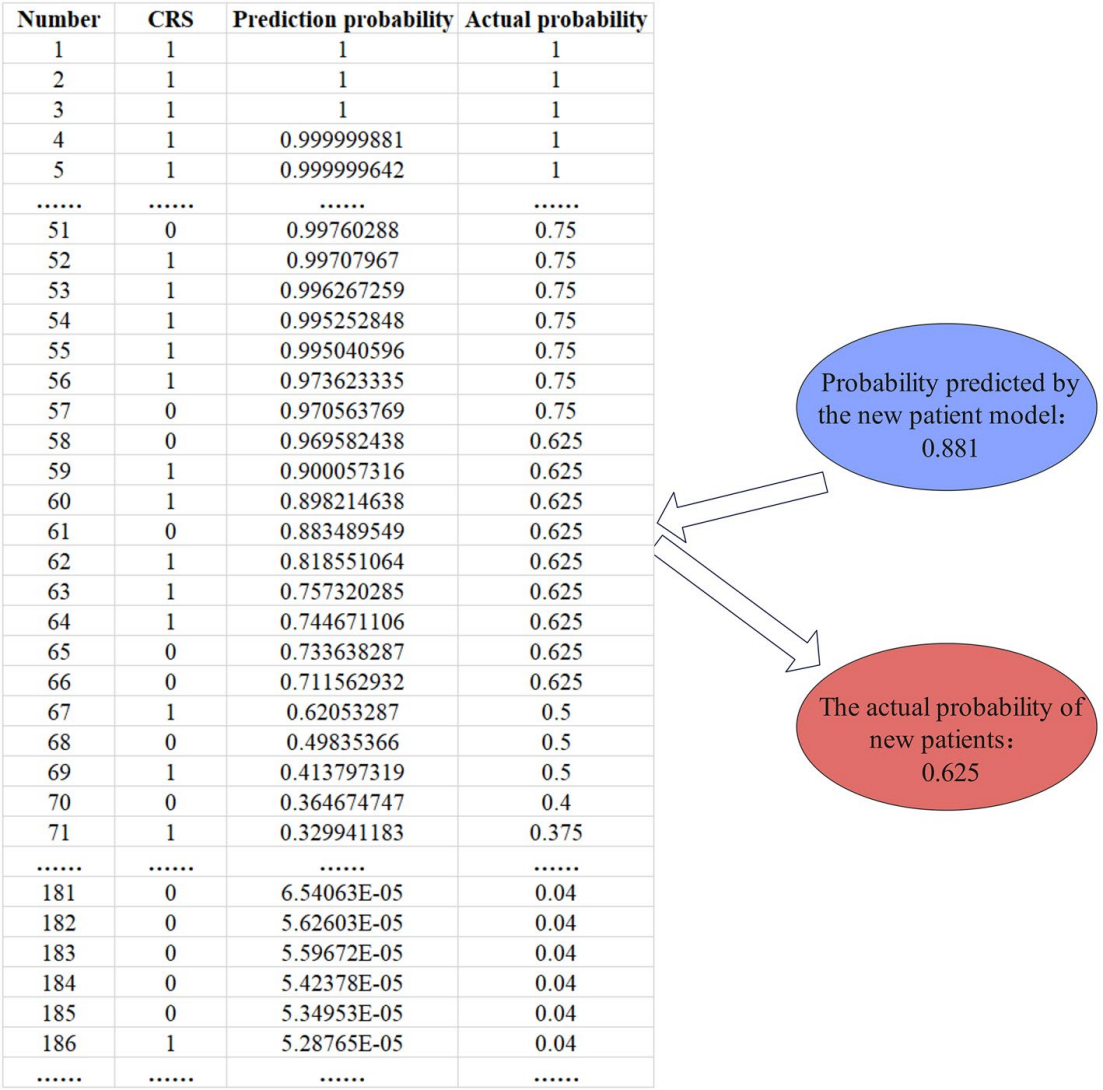
Diagram of the transformation between the predicted probability of the model and the actual probability. The figure uses 25 examples as illustrative examples, while there are others that follow but are not shown here for the sake of simplification

When the model is used to predict new cases and data, its prediction probability of the new data is matched to the saved baseline, and the position within the baseline that is closest to that probability is found. As shown in Fig. 5, for a new patient, the model predicted a probability of 0.881 for severe CRS. In the previously saved baseline data, position No. 60 corresponds to a probability of 0.898, position No. 61 corresponds to a probability of 0.883, position No. 62 corresponds to a probability of 0.819. Therefore, the position closest to the target probability of 0.881 is the probability value corresponding to the position No. 61 in the benchmark data. After the corresponding location is found, approximately 10 sample ranges are selected from the vicinity (above and below) of the location. If the corresponding position is in the front and there is not enough data in the front (less than 10), then select all the available data in the front and select 5–10 as the data range in the back. The number of CRS ≥ 3 in the selected sample is added and then divided by the selected sample range to obtain a representation of the actual probability.

Within the baseline range we recorded, the severity of CRS occurrence in each sample was recorded according to actual patients, and the ranking was arranged according to the best prediction probability during model training. In order to improve fault tolerance, the ratio of the number of CRS ≥ 3 in the selection range to the selection range was calculated. In our data, the number of CRS ≥ 3 was small, so we chose 10 as a suitable range value. At the same time, because the number of patients with severe CRS is relatively small compared with those without severe CRS, the number of samples corresponding to each 0.1 probability range is not large when the probability is above 0.8 within the baseline range. Therefore, when the probability exceeds 0.8, it is appropriate to choose 10 as the upper and lower interval value range. For our data, when the probability range is 0.3–0.8, the number of samples within each 0.1 probability range shows an increasing trend, so 15 can be selected as the value range of the upper and lower ranges. When the probability is lower than 0.3, the number of samples in each 0.1 probability range is the largest, so we choose 20 as the value range of the upper and lower intervals.

It is necessary to comprehensively consider the number of samples and the prediction probability distribution of the model to determine how many ranges to select as an interval. From a theoretical point of view, the greater the number of selected ranges, the stronger the actual probability tolerance, and the obtained probability estimate is closer to the actual probability of patients with severe CRS. Therefore, we believe that the probability representation obtained by this treatment is an approximate estimate of the actual probability of severe CRS occurrence.

**Fig. 6:**
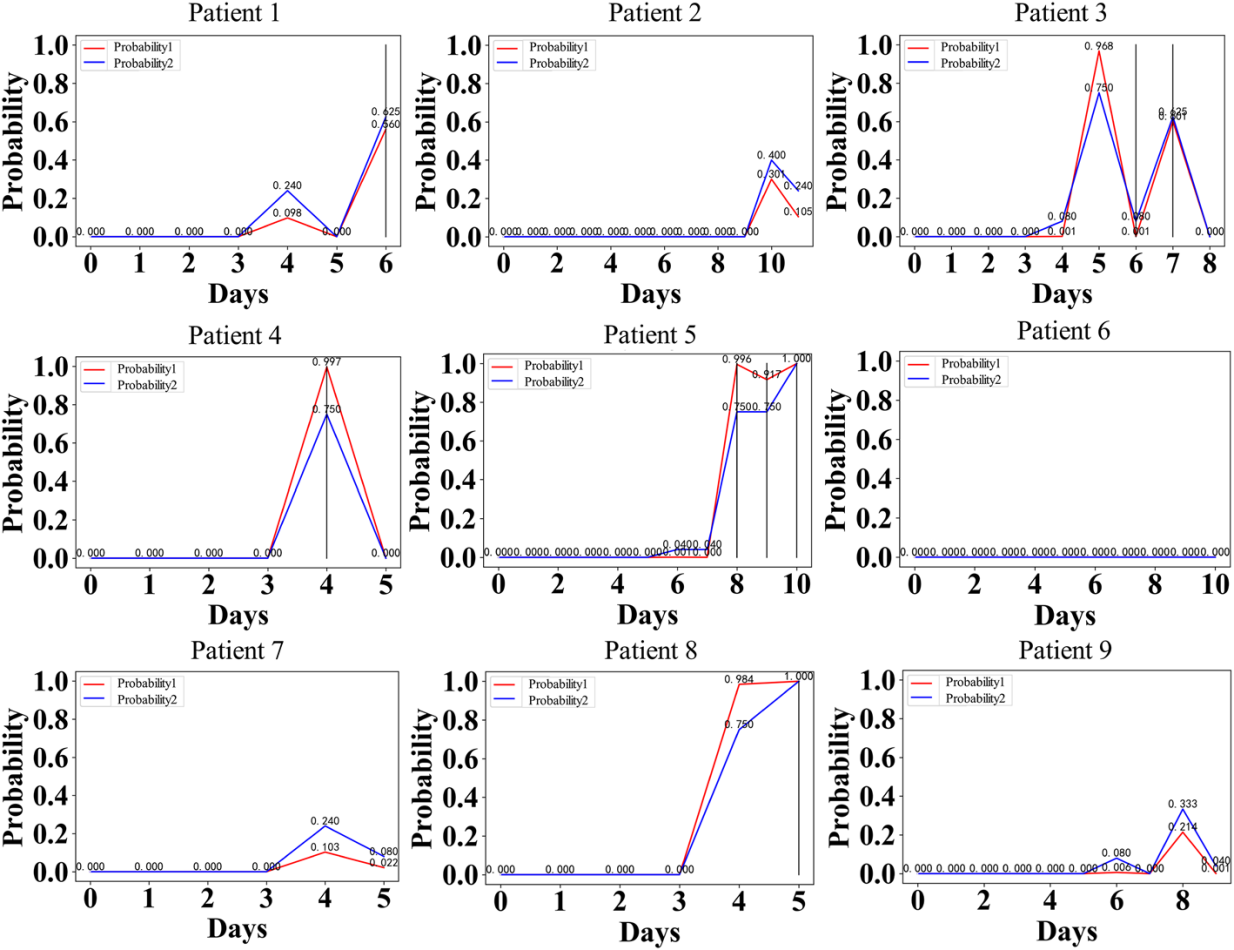
Verification chart of patients: a total of 9 patients were verified, with 5 experiencing severe CRS and 4 not. Two types of probabilities are utilized to represent the model's prediction status, and the probability indicated by the blue curve labeled as ‘Probability 2’ aligns more closely with actual patient outcomes

The actual probability is depicted by a blue line in the diagram, denoted as "Probability 2", while the initial model-generated probability is represented by a red line, labeled as "Probability 1", as illustrated in Fig. 6. The actual probability deviates slightly from the model's predicted probability, exhibiting a reduced frequency at both extremes. This pattern aligns more closely with the actual patient scenario and enhances diagnostic accuracy for medical practitioners.

**Fig. 7:**
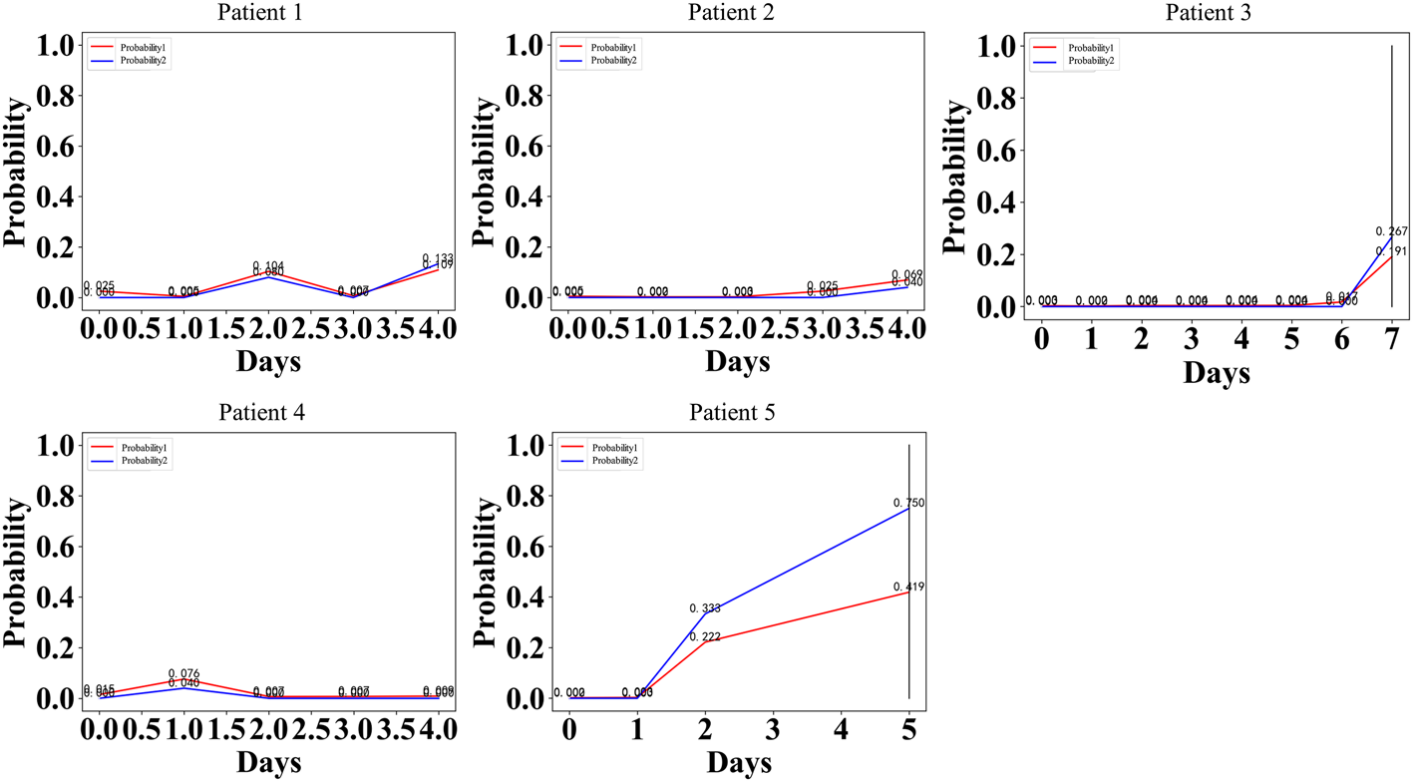
Patient validation map: 5 patients from other hospitals were used for further validation. 2 patients developed severe CRS and 3 patients did not develop severe CRS

As a result of our research, we realized that using data from one hospital and a relatively small number of patients in China could introduce bias and limit the applicability of the findings to the wider population. However, in order to ensure the universality and real-world applicability of the evaluation model, we conducted a second additional validation, selecting 5 patients from the Third Xiangya Hospital of Central South University as samples. According to the verification results (see Fig. 7), it can be seen that the model has good generalization ability.

### PrCRS reveals the influence of different clinical factors on human body

**Fig. 8:**
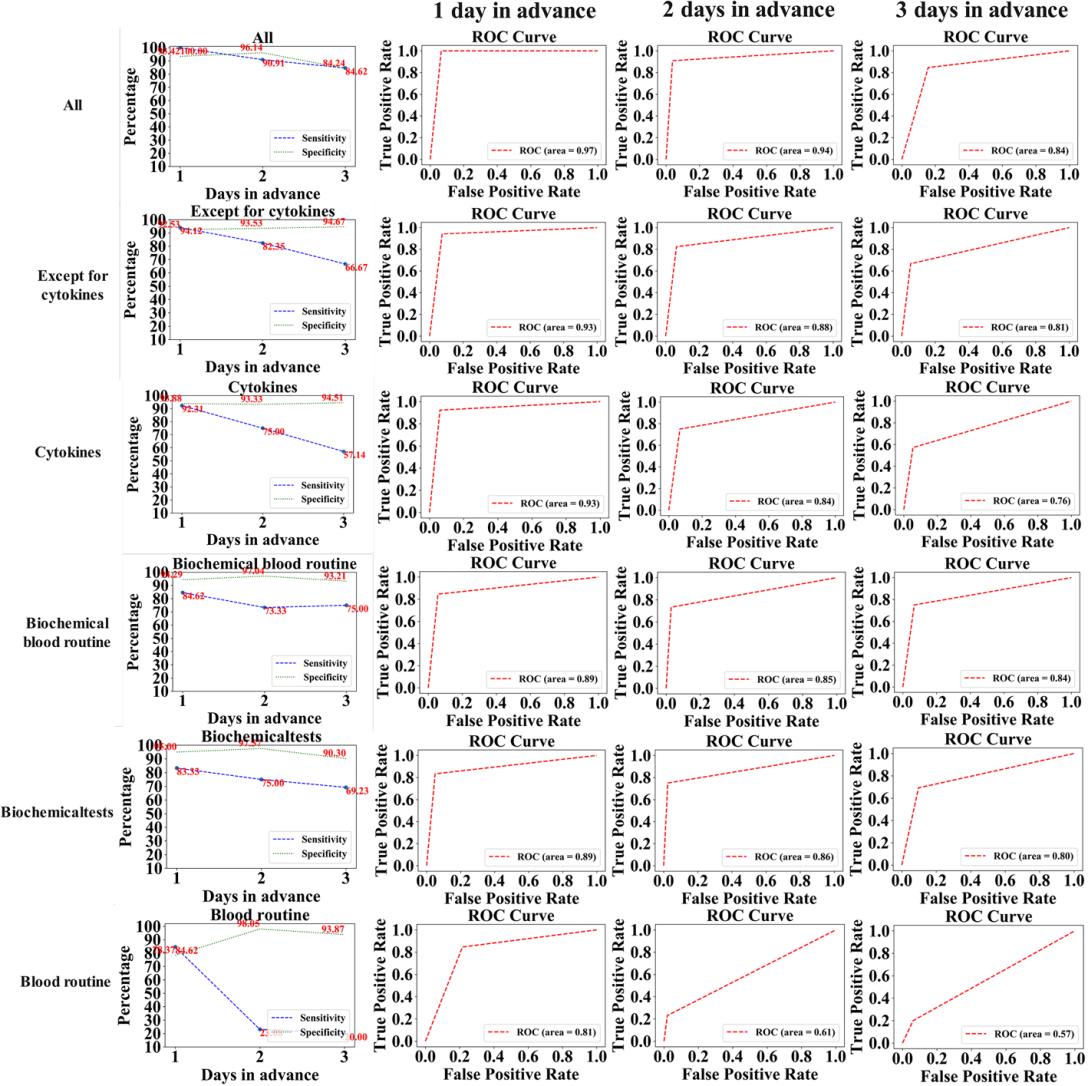
The predictive performance of the six models for patients at one, two, and three days in advance is illustrated using ROC curves and line charts

Subsequently, we conducted an analysis on the predictive model utilizing the complete patient dataset of 202 cases to forecast patient progression by one, two, and three days in advance. To evaluate the efficacy of each method at different time intervals, experimental validations were performed. The ROC curves depicting prediction results for each model at various time points and corresponding line charts illustrating changes are presented in Fig. 8. The specificity and sensibility of the prediction results decrease with an increase in the number of days in advance for all models, while maintaining overall convergence. Notably, among all factor models considered, they exhibit the highest levels of sensitivity and specificity. Although the lead time is decreasing, overall, the previous models still exhibit sensitivity and specificity rates above 50% and 90%, respectively, on the third day; above 80% and 95% if predicted one day in advance; and above 65% and 90% if predicted two days in advance. The overall predictive effect holds significant guidance for medical practitioners. Furthermore, we present the prediction outcomes in the form of probability to assess the likelihood of severe CRS, enabling doctors to visually perceive patients' risk more intuitively.

The model achieved the highest sensitivity and specificity one day in advance. In the prediction hierarchy, cytokines exhibited a prominent role followed by biochemical items and blood routine analysis. This observation underscores the pivotal involvement of cytokines in the pathogenesis of severe CRS among patients, thereby establishing their hierarchical significance. A series of subsequent reactions and activated pathways also played a pivotal role. Previous studies have indicated that IL-6, released by macrophages and monocytes, appears to be the primary driving factor behind CRS [32]. Higher levels of cytokines can be observed in severe CRS [33]. The release of IL-1 from activated macrophages and monocytes stimulates the release of IL-6 and induces nitric oxide synthase, thereby contributing to vascular damage [34]. Additionally, elevated serum levels of IL-2, TNF-ι, IFN-, IL-8, IL-10, MCP-1 and MIP-1 released by CAR-T cells activate T cells and further exacerbate inflammation [35]. The release of pro-inflammatory mediators, such as nitric oxide (NO), interleukin-1β (IL-1β), interleukin-2 (IL-2), interleukin-6 (IL-6), and tumor necrosis factor-alpha (TNF-α) during chronic inflammation often triggers a diverse array of molecular signaling cascades, including NF-KB, MAPK, and JAK/STAT. These cascades subsequently initiate an amplification loop for cytokine production. Among them, NF-KB serves as a central regulator in multiple signaling pathways, orchestrating the activation of diverse genes and their corresponding products [36]. Moreover, it further amplifies the inflammatory response [37]. These observations collectively indicate that cytokines alone possess remarkable predictive accuracy for severe CRS occurrence, thereby establishing a solid scientific foundation.

Among the individual predictions, the second one involves utilizing biochemical markers such as C-reactive protein and ferritin, which exhibit a strong correlation with severe CRS prediction. These factors have significantly contributed to achieving high sensitivity and specificity in this context. The levels of CRP, serum ferritin, and D-dimer have been demonstrated to be associated with severe CRS [38]. However, this correlation exhibits a weaker magnitude compared to cytokines, and the subsequent cascade reaction is not as robust as that induced by cytokines.

The last prediction utilizes blood routine analysis, revealing that various factors in the blood composition, such as the count and percentage of different blood cells, exhibit limited predictive capability for severe CRS occurrence. Consequently, there exists a weak correlation resulting in low sensitivity and specificity of the prediction outcomes. Therefore, we recommend prioritizing combinations ranked at the forefront for forecasting purposes.

### Prediction of webpage content description

**Fig. 9:**
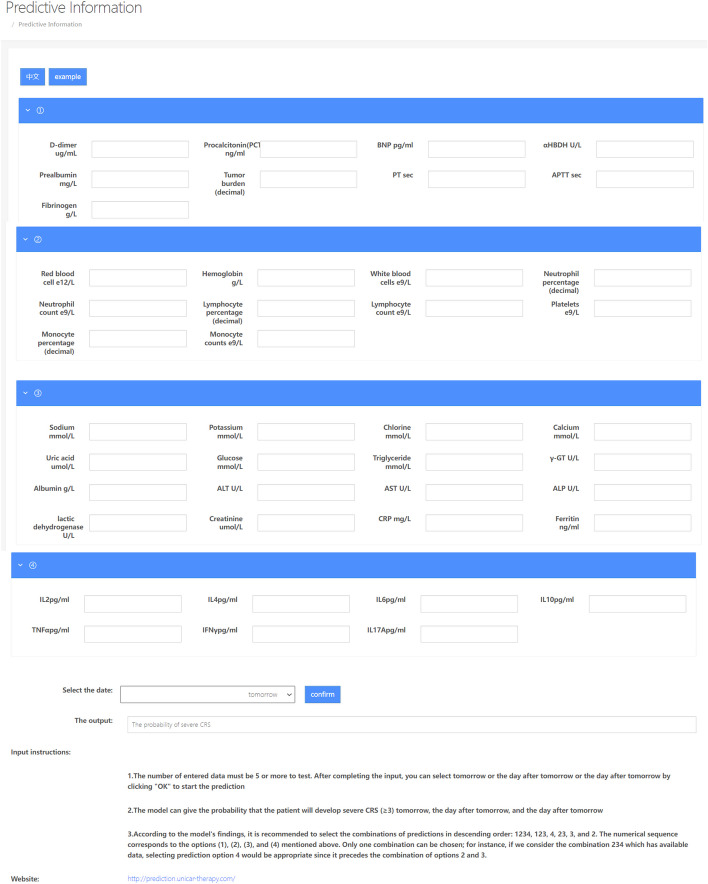
This webpage provides predictive analytics encompassing all essential clinical factors for patient treatment, categorized into six models, enabling doctors to assess the likelihood of patient progression within 1, 2, and 3 days

We have developed predictive models for blood routine, biochemical parameters, cytokines, and all clinical factors of patients to forecast severe CRS (probability ≥ 3) one day in advance, two days in advance, and three days in advance respectively. Additionally, we have designed a bilingual website for physicians to access these predictions at http://prediction.unicar-therapy.com/index-en.html. We present six models, wherein a minimum of five data inputs per page is required for accurate predictions. Following completion of the input process, users can select either tomorrow, the day after tomorrow, or the day thereafter to generate predictions. The predicted web interface is illustrated in Fig. 9. According to the model test results, we recommend selecting combinations for prediction in a descending order of significance. The suggested sequence of selection is as follows: 1234 (all variables), 123 (excluding cytokines), 4 (only cytokines), 23 (biochemical blood routine), 3 (only biochemical items), and 2 (only blood routine). Only one combination can be selected as input for forecasting. Among these combinations, the model takes a one-dimensional input vector (X_1_, X_2_, …, Xn), which represents the daily recorded clinical data of patients. The total number of factors identified was 42, including 9 factors related to coagulation and tumor load: D-dimer, procalcitonin, B-type natriuretic peptide, α-hydroxybutyrate dehydrogenase, prealbumin, primitive cells (tumor load), plasma prothrombin time, activated partial prothrombin time, and fibrinogen. The blood routine analysis includes 10 factors: red blood cell count, hemoglobin level, white blood cell count, neutrophil percentage and count, lymphocyte count, platelet count, monocyte percentage and count. The panel of biochemical factors in 3 includes sodium, potassium, chlorine, calcium, uric acid, glucose, triglyceride, γ-glutamyl transpeptidase, albumin, alanine aminotransferase (ALT), aspartate aminotransferase (AST), alkaline phosphatase (ALP), lactate dehydrogenase (LDH), creatinine, C-reactive protein (CRP) and ferritin.

## Discussion

The advent of CAR-T cell immunotherapy has revolutionized biomedical research, yet the emergence of cytokine release syndrome (CRS) during treatment poses a significant threat to patient safety. Currently, there is an absence of deep learning-based prediction models for accurately forecasting the timing and probability of CRS prevention. The limited number of patients undergoing CAR-T therapy poses a significant bottleneck, while the deep learning model necessitates a larger dataset. Currently, a decision tree model based on machine learning is employed for predicting the occurrence of severe CRS. The drawback of this approach lies in its limited flexibility compared to deep learning, resulting in suboptimal prediction accuracy when utilizing branches of the model tree. To address this limitation, we have developed PrCRS, a deep learning model, aiming to bridge this gap. Furthermore, we employ transfer learning techniques to compensate for the scarcity of data. The transferred data originates from COVID-19 patient records, enabling automated prediction of the likelihood of severe CRS occurrence in patients at least one day in advance. This facilitates timely assessment by medical professionals upon inputting new patient data.

In the learning phase, we employ a combination of U-Net and Transformer architectures, along with employing transfer learning techniques. Based on the evaluation results, our model demonstrates superior efficiency compared to the state-of-the-art models with equivalent parameter quantities. The learning ability of our model is robust, and the incorporation of a multi-attention module endows it with parallel computing capabilities. This significantly reduces computational overhead and enhances its proficiency in predicting severe CRS occurrences. Based on this foundation, we construct six distinct forecasting models incorporating various factors and provide ranking recommendations based on the sensitivity and specificity indicators obtained from the test dataset. We developed a web-based platform and implemented a model output strategy that transforms the predicted occurrence of severe CRS into probability values, facilitating timely patient assessment by healthcare professionals up to three days in advance.

The PrCRS model has been trained and tested using our proprietary datasets. Although the evaluation results demonstrate its excellent performance, there are still instances where patients with severe CRS exhibit relatively low probabilities, while certain non-severe CRS cases show high probabilities. Moreover, we conducted independent verification on 9 patients and further validated the model's performance on this dataset by retraining it with a cohort of 193 patients. These limitations can be addressed in future research through the utilization of advanced network models, additional data validation techniques, and an expanded training dataset.

We have recently released an open-source PrCRS platform, encompassing both Chinese and English versions, along with the corresponding open-source code available on GitHub (https://github.com/wzy38828201/PrCRS). The repository comprises comprehensive source code, as well as detailed instructions and scripts for the examples presented in this article. The repository also includes the network training module, enabling further model refinement to enhance the performance of untested applications. Additionally, comprehensive training and test datasets are provided. Our CRS analysis platform serves as a pivotal tool for deep learning models in CAR-T research. The platform is capable of generating probability estimates even in the absence of complete data. We have developed six models, enabling the input of various types of measurement data related to cytokines, biochemical markers, and blood routine for accurate assessments. The platform can be extended to a range of severe CRS judgment scenarios without requiring parameter adjustment, thereby highlighting the platform's potential in standardizing the determination of severe CRS and enhancing reproducibility. PrCRS enables comprehensive and convenient analysis of severe CRS, facilitating faster and more accurate patient assessment, thus contributing to medical system research.

## Conclusion

The PrCRS system enables comprehensive and convenient analysis of severe CRS, facilitating faster and more accurate patient assessment. It has been utilized in medical research to enhance the efficiency of healthcare systems. Moreover, the model serves as a valuable source of inspiration for developing a neural network-based CRS prediction model in CAR-T therapy. Additionally, the concept of transfer learning can be seamlessly applied to other methodologies, thereby offering a comprehensive approach for optimizing Transformer-type prediction methods based on deep learning.

## Data Availability

Source code URL: https://github.com/wzy38828201/PrCRS. Website address: http://prediction.unicar-therapy.com/index-en.html.
